# Methanol Extract of *Pueraria lobata* (Willd.) Root and Its Active Ingredient, Puerarin, Induce Apoptosis in HeLa Cells and Attenuates Bacterial Vaginosis in *Gardnerella vaginalis*-Infected Mice

**DOI:** 10.3390/ijms26031342

**Published:** 2025-02-05

**Authors:** Ji-Hyun Lee, Ji-Ye Lim, Yong-Deok Jeon, Dae-Ki Kim, Dong-Hyun Lee

**Affiliations:** 1 Department of Immunology, Medical School, Jeonbuk National University, Jeonju 54907, Republic of Korea; jihyunsh1211@naver.com (J.-H.L.); 84juce@naver.com (J.-Y.L.); 2 Department of Korean Pharmacy, Woosuk University, Wanju 55338, Republic of Korea; ydjeon1211jh@woosuk.ac.kr; 3 Department of Obstetrics and Gynecology, Jeonbuk National University Hospital, Jeonju 54907, Republic of Korea; 4 Research Institute of Clinical Medicine of Jeonbuk National University-Biomedical Research Institute of Jeonbuk National University Hospital, Jeonju 54907, Republic of Korea

**Keywords:** cervical cancer, *Pueraria lobata* root, puerarin, HeLa cells, *Gardnerella vaginalis*, vaginitis

## Abstract

*Pueraria lobata* (Willd.) has been used as food since ancient times, and its roots have been used mainly as a traditional herbal medicine to treat various diseases in East Asia. Puerarin is one of the major active ingredients in the roots of *P. lobata*. The purpose of this study was to examine the effects of the methanol extract of *P. lobata* roots (PRME) and puerarin on apoptosis in cervical cancer and inflammation-relieving effects in vaginitis. First, we prepared the PRME and confirmed the puerarin content of PRME through HPLC analysis. We performed a TUNEL assay, Hoechst 33342 staining, and western blotting using HeLa cells, a human cervical cancer cell line. Both the PRME and puerarin exhibited antiproliferative effects in HeLa cells by inducing apoptosis through the activation of the extrinsic death receptor and intrinsic mitochondrial pathways, thereby demonstrating their anticancer efficacy against human cervical cancer. Next, a mouse model of vaginitis induced by *Gardnerella vaginalis* (GV) infection was established by inoculating C57BL/6 mice with β-estradiol-3-benzoate and GV (1 × 10^8^ CFU). Histological analysis and PCR confirmed that the administration of PRME or puerarin to GV-infected mice alleviated reproductive tract vaginitis symptoms. Additionally, we confirmed that PRME or puerarin treatment decreased myeloperoxidase activity and reduced inflammation by regulating cytokines through the secretion of inflammatory mediators in mouse vaginal tissue. These results demonstrate that PRME and puerarin can be used as potential adjuvants or therapeutic agents with anticancer and anti-inflammatory properties to inhibit the progression of human cervical cancer and alleviate vaginitis.

## 1. Introduction

Cervical cancer is a common cancer in women worldwide, with a high incidence and mortality rate [1]. According to the 2023 Korea Central Cancer Registry, cervical cancer accounts for 1.1% of all cancers. The 5-year survival rate for localized cervical cancer is reported to be 92.4%, while for distant metastasis, it is 24.6%. Cervical cancer occurs mostly in middle-aged women in their forties and fifties, but recently, the incidence rate in women in their twenties and thirties has also increased significantly. In most cases, there are no symptoms at all in the early stages; symptoms appear after cancer has progressed to a certain extent, and in some cases, even advanced cancer may not have any symptoms, so it is important to receive regular gynecological examinations and pap smears. Accordingly, the National Health Insurance Service of Korea provides free cervical cancer screening every two years for women over the age of 20. Cervical cancer, which is mainly caused by viruses or bacteria, is also affected by various factors such as heredity, stress, and the environment [2]. Current treatment methods for cervical cancer include surgery, radiotherapy, and chemotherapy such as bevacizumab and pembrolizumab; however, these anticancer drugs are adjuvant therapy and have serious side effects such as fatigue, insomnia, nerve damage, and gastrointestinal issues [3]. Therefore, to improve the survival rate of patients with cervical cancer, it is necessary to establish a therapeutic strategy to identify new effective and safe treatment approaches.

According to the Korea Disease Control and Prevention Agency, more than 90% of cases in premenopausal women are bacterial vaginosis (BV), candidiasis, and trichomoniasis, and among these, BV is the most common, accounting for 40 to 50% of the total. BV is a common vaginal infection in women, characterized by a decrease in the number of protective bacteria in the vaginal flora and an increase in anaerobic bacteria, including *Gardnerella vaginalis* [4]. BV is associated with an increase in miscarriage, premature birth, and infertility; if the disease becomes chronic, it can cause various complications, such as pelvic inflammatory disease and cervicitis [5]. Additionally, it has been suggested that there may be a correlation between vaginitis and cervical cancer, as there have been cases where vaginitis was found in cervical cancer screening. Currently, antibiotics such as metronidazole and clindamycin are used as treatments for BV, but they have the disadvantages of adverse effects such as indigestion, gastrointestinal disorders, headaches, and liver abnormalities when taken for a long period and a high possibility of BV recurrence due to resistance [6]. Because of the lack of appropriate treatments for BV, there has been an increasing interest in administering probiotics to improve BV while avoiding the side effects of antibiotics [7]. However, the effects of single-strain probiotics are insufficient for treating BV [8]. Therefore, it is necessary to identify a safe substance that can be used as an additional supplement or new treatment to alleviate BV.

Recent studies have reported that medicines containing natural products have fewer adverse effects than chemically synthesized medicines and are more appropriate for long-term use. Therefore, we focused on the antitumor and anti-inflammatory properties of these natural products.

The root of *Pueraria lobata* (Willd.) has been traditionally used to treat headaches, diarrhea, fever, and detoxification and has also been commercialized as a Western dietary supplement [9]. The roots of *P. lobata* (Willd.) contain active ingredients such as puerarin, daidzin, and daidzein, with puerarin present in significant amounts [10]. Puerarin is one of the isoflavones found in plants. It has the chemical formula C_21_H_20_O_9_ and is composed of two benzene rings with an oxygen atom bonded between them. Puerarin is a multifaceted compound with a wide range of pharmacological activities. Puerarin is known to have antioxidant and anti-inflammatory activities, as well as cardiovascular, hypoglycemic, and antiplatelet aggregation effects. In addition, puerarin has been confirmed to have therapeutic effects on many diseases, such as diabetes, liver damage, dermatitis, and colitis [11]. We have been conducting pharmacological studies using the root of *P. lobata* (Willd.) and puerarin for many years. We aimed to elucidate the basic mechanisms of the pharmacological activity of the root of *P. lobata* (Willd.) and puerarin and explore their potential for treating other diseases. Although many studies have been conducted on extracts of *P. lobata* (Willd.) and puerarin, to date, studies on their anticancer effects in cervical cancer or their anti-inflammatory effects in vaginitis are lacking.

The purpose of this study was to investigate the potential anticancer and anti-inflammatory effects of the extracts of the root of *P. lobata* (Willd.) and its active ingredient, puerarin, to discover new natural therapeutic candidates for alleviating cervical cancer and vaginitis.

## 2. Results

### 2.1. Active Components Analysis and Quantification of PRME Extract by HPLC

**Figure 1:**
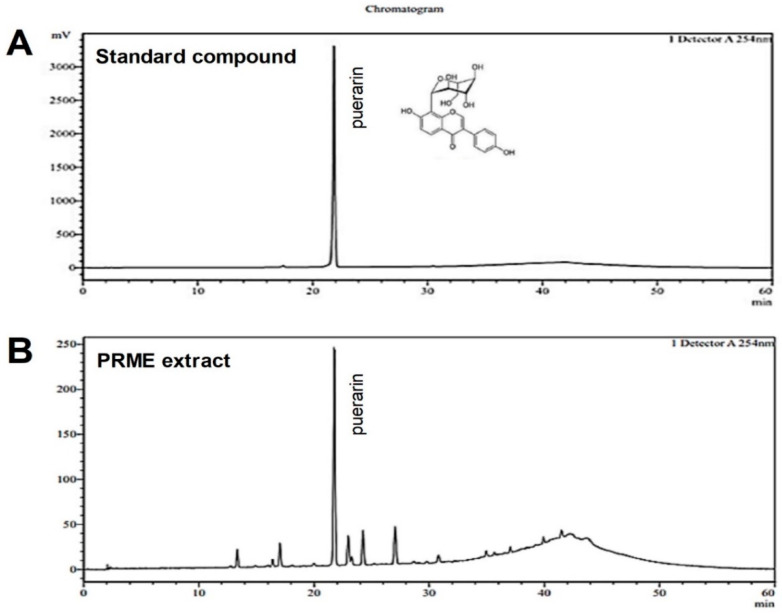
HPLC analysis of the PRME extract. HPLC chromatograms of (**A**) the puerarin standard compound and (**B**) the PRME extract.

The content of puerarin, the main component of the PRME extract, was analyzed using HPLC with a UV-VIS detector (Figure 1). The puerarin peak in the PRME extract was confirmed by comparison with that of the internal standard component. The puerarin concentration in the PRME extract was 0.081 mg/g.

### 2.2. PRME Extract and Puerarin Attenuate Proinflammatory Cytokine Levels in HeLa Cells

**Figure 2:**
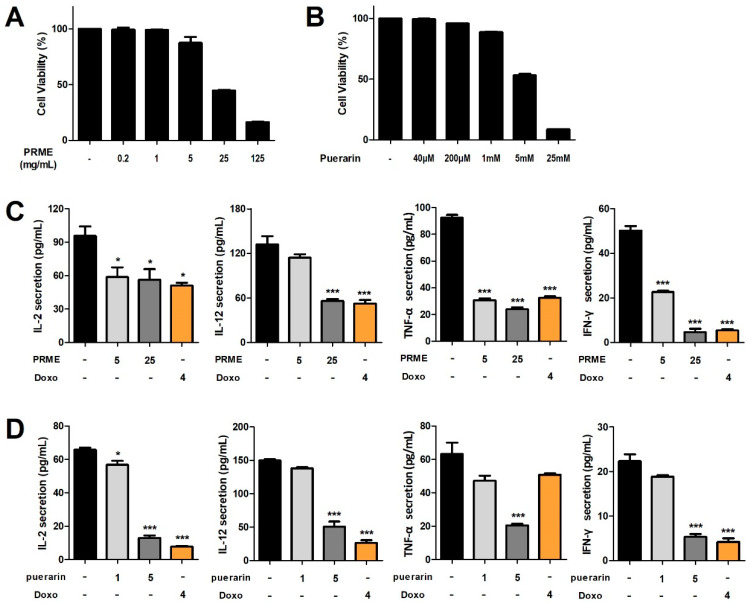
Effects of the PRME extract and puerarin on cell viability and proinflammatory cytokine levels in HeLa cells. (**A**,**C**) Cell viability and the proinflammatory cytokine levels of the PRME extract. (**B**,**D**) Cell viability and the proinflammatory cytokine levels of puerarin. Data are presented as the mean ± SEM of the three experiments. * *p* < 0.05 or *** *p* < 0.001 compared to the nontreatment condition. IL-2: interleukin-2, IL-12: interleukin-12, IFN-γ: interferon-γ, TNF-α: tumor necrosis factor-α, and Doxo: doxorubicin.

We investigated the cell viability of PRME extract and its main active ingredient, puerarin, on HeLa cells using the MTT assay. We confirmed that cell viability decreased as the dose of PRME extract and puerarin increased. Thus, the administration of PRME extract for 24 h resulted in cell viability of 87.44% at 5 mg/mL and 44.73% at 25 mg/mL (Figure 2A). In addition, the administration of puerarin for 24 h resulted in cell viability of 87.89% at 1 mM and 49.22% at 5 mM (Figure 2B). After setting the treatment concentrations of PRME extract and puerarin based on the cell viability results, we examined the effects of PRME extract and puerarin on inflammatory cytokine levels in HeLa cells. We established that cytokine levels comprising IL-2, IL-12, IFN-γ, and TNF-α were all reduced in HeLa cells treated with the PRME extract or puerarin, and in particular, a significant decrease was observed at high concentrations of PRME extract and puerarin (Figure 2C,D).

### 2.3. PRME Extract and Puerarin Induce Apoptosis in HeLa Cells

**Figure 3:**
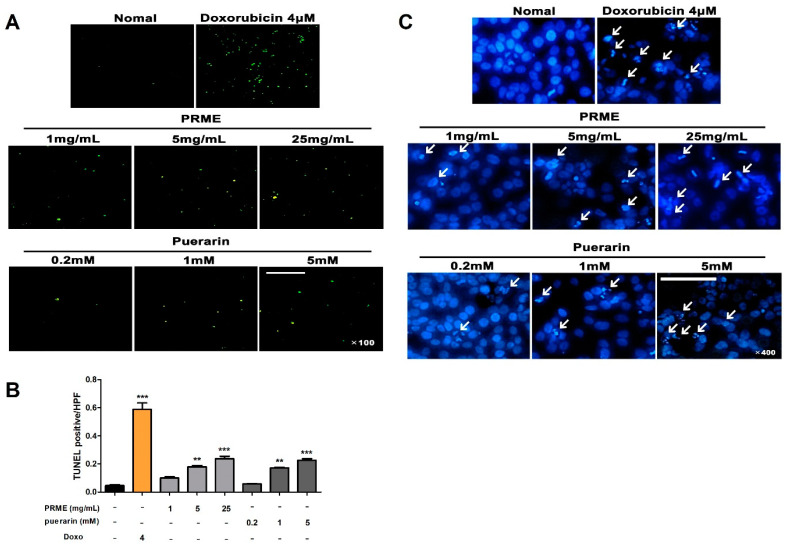
Effect of the PRME extract and puerarin on apoptosis in HeLa cells. Typical nuclear morphological changes of apoptosis in HeLa cells were detected by staining with (**A**) the TUNEL and (**C**) Hoechst 33,342 assay kits. Representative staining images were taken with a fluorescence microscope (TUNEL assay: scale bar = 40 μm, 100×; Hoechst 33,342 assay: scale bar = 100 μm, 400×). White arrows point to apoptotic cells. (**B**) The fluorescence intensity by the TUNEL assay staining is represented as a bar graph. Data are shown as the mean ± SEM of the three experiments. ** *p* < 0.01 or *** *p* < 0.001 compared to the nontreatment condition.

To examine the apoptotic activity of the PRME extract and puerarin, we treated HeLa cells with samples at different concentrations and analyzed them using TUNEL and Hoechst 33,342 apoptosis detection kits. Apoptotic HeLa cells showed round and condensed cell bodies, as indicated by white arrows. As shown in Figure 3, apoptosis increased with increasing concentrations of both PRME extract and puerarin in HeLa cells, suggesting that apoptosis induced by PRME extract and puerarin contributed to the decrease in the cell viability of HeLa cells.

### 2.4. PRME Extract and Puerarin Activate Apoptosis-Related Genes in HeLa Cells

**Figure 4:**
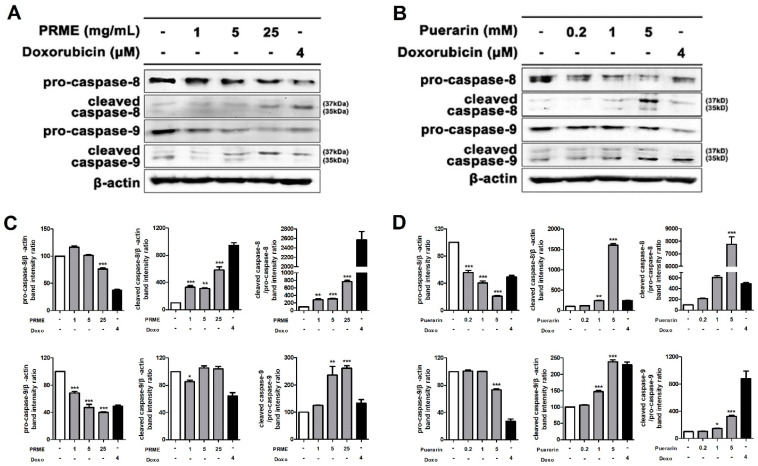
Effect of the PRME extract and puerarin on apoptosis-related protein expression in HeLa cells. (**A**,**B**) Regulation protein expression levels of apoptosis-related genes (pro-caspase-8, cleaved caspase-8, pro-caspase-9, and cleaved caspase-9) by the PRME extract and puerarin in HeLa cells, analyzed by western blotting. (**C**,**D**) The bar graphs for the relative density of protein bands from western blots are as follows: pro-caspase-8/β-actin, cleaved caspase-8/β-actin, cleaved caspase-8/pro-caspase-8, pro-caspase-9/β-actin, cleaved caspase-9/β-actin, and cleaved caspase-9/pro-caspase-9. Data are shown as the mean ± SEM of the three experiments. * *p* < 0.05, ** *p* < 0.01 or *** *p* < 0.001 compared to the nontreatment condition.

To confirm the apoptotic mechanism of the PRME extract or puerarin treatment in HeLa cells, the expression of apoptosis-related proteins was examined by western blotting. Figure 4 shows the effects of PRME and puerarin on caspase-8 and caspase-9, which are involved in the extrinsic and intrinsic apoptotic pathways, respectively. The protein expression of pro-caspase-8 and pro-caspase-9 was dose-dependently decreased by PRME and puerarin treatment in HeLa cells, whereas that of the cleaved versions of caspase-8 and -9 increased. These results imply that the PRME extract and puerarin play an important role in inducing apoptosis in HeLa cells by activating apoptosis-related genes, supporting their potential as anticancer drugs.

### 2.5. PRME Extract and Puerarin Alleviate Vaginitis Symptoms and MPO Activity in GV-Infected Mice

**Figure 5:**
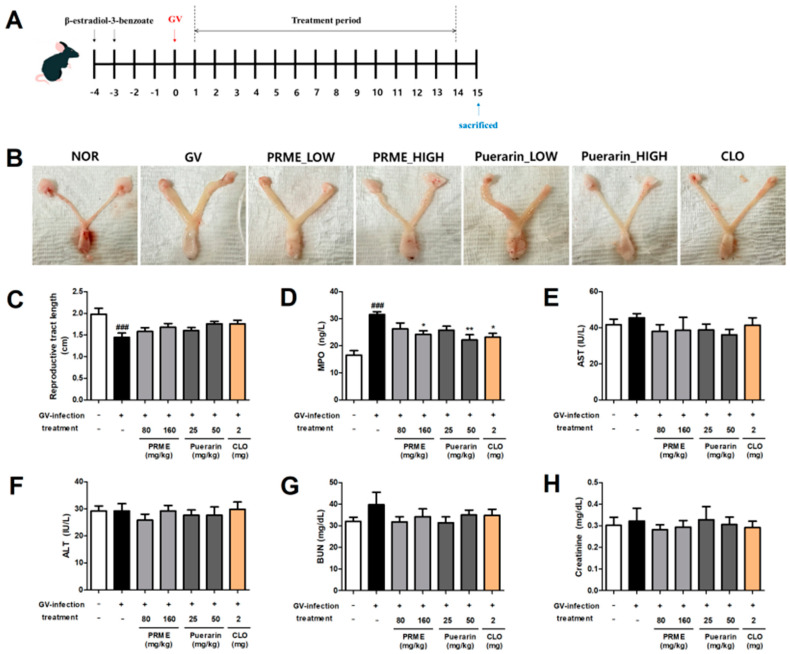
Effect of the PRME extract and puerarin on vaginitis symptoms, MPO activity, and serum levels in GV-infected mice. (**A**) Experimental design for the GV-infected vaginitis mice model. (**B**) Representative photographs of lesions of the reproductive tract (vagina and uterine horn) in mice from each group. (**C**) Total length of the reproductive tract. (**D**) MPO activity in vaginal tissue. (**E**–**H**) Serum levels of aspartate aminotransferase (AST), alanine aminotransferase (ALT), blood urinary nitrogen (BUN), and creatinine. Data are the mean ± SEM of the three experiments. ^###^ *p* < 0.001 versus the normal group; * *p* < 0.05 and ** *p* < 0.01 versus the GV-infected group.

The experimental design used to induce vaginitis by infecting mice with GV is shown in Figure 5A. A classical sign of vaginitis is edema, and in this study, we observed an increase in the overall thickness of the reproductive tract in GV-infected mice due to edema. This edema was reduced in the treatment groups; in particular, significant decreases in reproductive tract thickness were observed in the high-concentration treatment groups (Figure 5B). The overall uterine length of GV-infected mice was dramatically reduced compared to that of the normal group. In contrast, all treatment groups showed less reduction in the overall uterine length than the GV group, but the change was not significant (Figure 5C). MPO activity was assessed using vaginal tissue lysates. MPO activity induced by GV infection was increased by 91.12% compared to the normal group and was confirmed to be reduced by 16.79% by the low-concentration PRME extract treatment and 23.13% by the high-concentration PRME extract treatment. In addition, it was confirmed that the treatment with low concentration of puerarin decreased by 18.33%, the treatment with high concentration of puerarin decreased by 29.68%, and the treatment with CLO, which is a positive control, decreased by 26.51% (Figure 5D). We evaluated the biochemical parameters (ALT, AST, BUN, and creatinine) to determine whether GV infection and treatment with the PRME extract or puerarin resulted in signs of toxicity in mice. As a result, it was confirmed that no group treated with PRME extract or puerarin showed a significant increase in the values of biochemical parameters compared to the normal group (Figure 5E–H). This indicates that the treatment with PRME extract or puerarin does not cause toxicity in mice.

### 2.6. PRME Extract and Puerarin Restore Histopathological Changes in GV-Infected Mice

**Figure 6:**
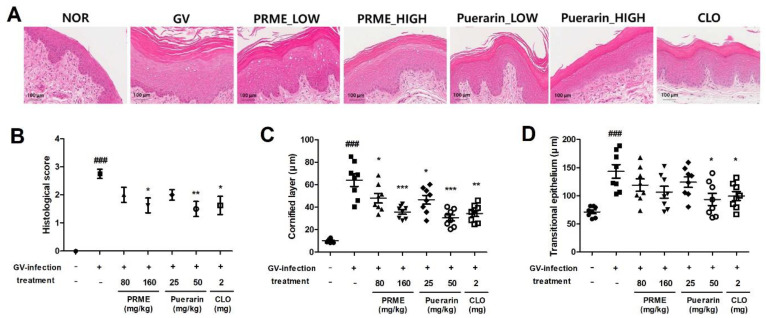
Effect of the PRME extract and puerarin on histological changes in mice with GV-induced vaginitis. (**A**) Representative images of histological changes in the vaginal confirmed by H&E staining (×200 magnification). (**B**) Histological score. (**C**) Vaginal stratum corneum (cornified layer) and (**D**) transitional epithelium thickness. Data are the mean ± SEM of the three experiments. ^###^ *p* < 0.001 versus Normal group; * *p* < 0.05, ** *p* < 0.01, and *** *p* < 0.001 versus the GV-infected group.

The major clinical features of vaginitis include vaginal epithelial cell exfoliation (stratification), transitional epithelial cell proliferation, and inflammatory cell infiltration [12]. Therefore, we examined the histopathological changes in the vaginal tissues of each group using H&E staining to evaluate the effects of PRME extract and puerarin in GV-infected mice. As shown in Figure 6A, a significant increase in the cornified layer and transitional epithelial thickness due to the proliferation of epithelial cells, stratification of the epithelium, and infiltration of inflammatory cells were observed in the vaginal tissues of GV-infected mice compared to the normal group. However, all treatment groups showed symptom restoration compared to GV-infected mice. Epithelial damage was significantly reduced by treatments with high concentrations of the PRME extract or puerarin. In particular, treatments with high concentrations of puerarin showed a more effective restorative effect than treatments with the positive control, CLO. Consistent with our previous data, the histological score (severity of exfoliation and inflammation of the vaginal epithelial lesions) was significantly increased in GV-infected mice but showed a tendency to decrease in all treatment groups and was considerably reduced by treatments with high concentrations of the PRME extract or puerarin (Figure 6B). The cornified layer and transitional epithelium thickness was increased by GV infection, and showed recovery in all treatment groups, the most effective group being the high-concentration puerarin group (Figure 6C,D).

### 2.7. PRME Extract and Puerarin Suppresses the Expression of COX-2 and iNOS in GV-Infected Mice

**Figure 7:**
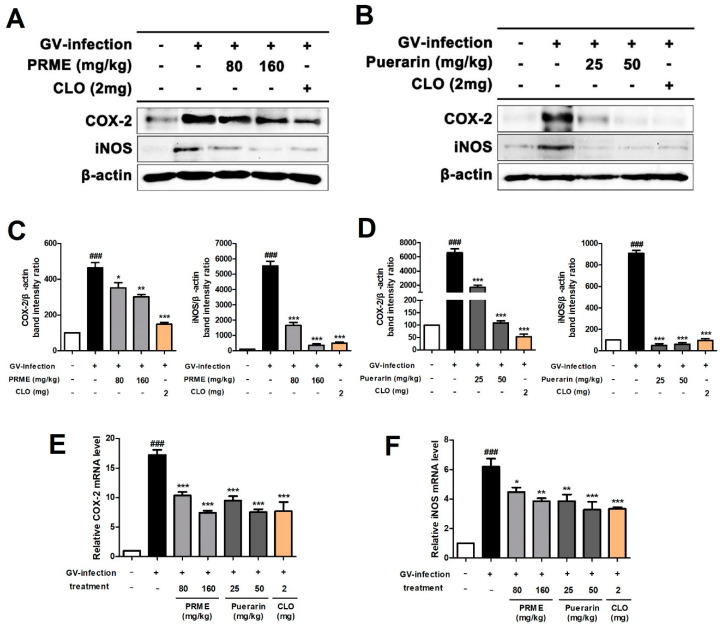
Effect of the PRME extract and puerarin on the expression of COX-2 and iNOS in mice with GV-induced vaginitis. (**A**,**B**) Regulation of COX-2 and iNOS protein expression by PRME extract and puerarin in mouse vaginal tissue analyzed by western blotting. (**C**,**D**) The bar graphs for the relative density of protein bands from western blots are as follows: COX-2/β-actin and iNOS/β-actin. (**E**,**F**) mRNA expression of COX-2 and iNOS in mouse vaginal tissue analyzed by RT-qPCR. Data are the mean ± SEM of the three experiments. ^###^ *p* < 0.001 versus Normal group; * *p* < 0.05, ** *p* < 0.01, and *** *p* < 0.001 versus the GV-infected group.

COX-2 and iNOS, which play an important role in mediating inflammatory responses and regulating cytokine levels, are expressed more when inflammatory responses occur due to external stimuli. Western blotting analysis was conducted to investigate whether COX-2 and iNOS are involved in the anti-inflammatory mechanism of PRME extract and puerarin in GV-infected mice. We found that the protein expression of COX-2 and iNOS was upregulated following GV infection. However, the increased protein expression of COX-2 and iNOS was effectively downregulated by the oral administration of the PRME extract or puerarin (Figure 7A–D). Similarly, the increased mRNA expression of COX-2 and iNOS in the vaginal tissues of GV-infected mice was significantly inhibited in a dose-dependent manner by the oral administration of the PRME extract or puerarin (Figure 7E,F). These results suggest that inflammation is suppressed by treatment with PRME extract or puerarin in GV-infected mice.

### 2.8. PRME Extract and Puerarin Regulates the Cytokine Levels in GV-Infected Mice

**Figure 8:**
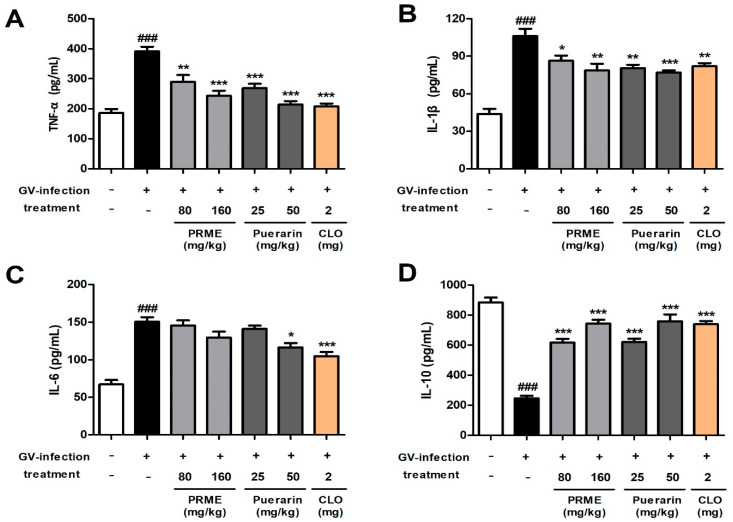
Effect of the PRME extract and puerarin on the inflammation-related cytokine levels in mice with GV-induced vaginitis. (**A**–**D**) Proinflammatory (TNF-α, IL-1β, and IL-6) and anti-inflammatory (IL-10) cytokine levels in vaginal tissue lysates from mice. Data are the mean ± SEM of the three experiments. ^###^ *p* < 0.001 versus the Normal group; * *p* < 0.05, ** *p* < 0.01, and *** *p* < 0.001 versus the GV-infected group.

To determine the effects of the PRME extract and puerarin on the release of cytokines (TNF-α, IL-1β, IL-6, and IL-10) in vaginal tissue lysates, ELISA was used to measure their levels. The levels of inflammatory cytokines, including TNF-α, IL-1β, and IL-6, in vaginal tissue lysates were significantly upregulated in the GV infection group compared to the normal group. However, inflammatory cytokines (TNF-α and IL-1β) induced by GV infection were significantly downregulated in all treatment groups orally administered with PRME extract and puerarin (Figure 8A,B). The increased level of IL-6 induced by GV infection was reduced by the oral administration of PRME extract and puerarin; however, a significant decrease was observed only in the high-dose puerarin group (Figure 8C). The level of IL-10, an anti-inflammatory cytokine, was reduced in the vaginal tissue lysates from GV-treated mice compared to that in the normal group and was significantly restored in a dose-dependent manner by oral administration of the PRME extract or puerarin (Figure 8D). Our results indicate that the PRME extract and puerarin exert anti-inflammatory effects by inducing a decrease in inflammatory cytokines and an increase in anti-inflammatory cytokines in GV-infected mice.

## 3. Discussion

The incidence of uterine diseases, common in middle-aged women, has been steadily increasing in young women. Therefore, the 2019 Korea Health Insurance Review and Assessment Service announced that ‘regular checkups are essential as the number of patients with uterine diseases among the young is increasing’. The reason why uterine diseases are increasing is because the period of exposure of the uterus to female hormones is getting longer as menarche is getting earlier and childbirth experience is getting later or less. As these uterine diseases can have serious consequences if neglected, continuous management for prevention and early treatment is important. Therefore, we aimed to identify natural plant-derived candidate substances with few side effects and good efficacy for the prevention and treatment of cervical cancer and vaginitis, which are representative uterine diseases.

In this study, we used HeLa cells and GV-infected mice to investigate the anticancer and anti-inflammatory effects of PRME extract and puerarin, its main ingredient. In a preliminary experiment, we quantified the puerarin content in *P. lobata* root extracts using various extraction solvents and determined that the highest amount of puerarin was detected in the methanol extract. The methanol extract was used in the subsequent experiments reported here.

In cancer cells, such as HeLa cells, various inflammatory cytokines are increased to continuously maintain tumor development and proliferation, inducing metastasis and angiogenesis within the tumor microenvironment [13]. Doxorubicin, which we used as a positive control in our in vitro experiment, is an anticancer drug that has been used in clinical practice for decades. Doxorubicin has been effective in slowing the progression of cancer, but it has the side effect of causing systemic toxicity in patients when used in high doses [14]. We first investigated the anti-inflammatory effects of PRME extract and puerarin in HeLa cells. As a result, both PRME extract and puerarin showed a pattern of reducing various proinflammatory cytokine levels, including IL-2, IL-12, IFN-γ, and TNF-α, in HeLa cells, thereby proving that the PRME extract and puerarin also have anti-inflammatory effects in human cervical cancer cells.

Apoptosis is a process of physiological cell death that is programmed to regulate the balance between cell survival and death [15]. Therefore, inducing apoptosis in cancer cells without affecting normal cells is a key mechanism in anticancer therapy. There are two distinct apoptotic signaling pathways: the extrinsic death receptor pathway and the intrinsic mitochondrial pathway [16]. The extrinsic death receptor pathway involves the family of Fas and TNF receptors, which activate caspase-8. The intrinsic pathway activates caspase-9 via cytochrome C released from the mitochondria due to mitochondrial dysfunction. These activated caspases then activate caspase-3, which drives apoptosis [17,18]. Our data showed that the PRME extract and puerarin led to apoptosis in HeLa cells, as confirmed by DNA fragmentation using the TUNEL assay and cell morphological changes (cell shrinkage and chromatin condensation) using Hoechst 33,342 staining. In addition, we confirmed through Western blot results that both the PRME extract and puerarin activated caspase-8 and -9 in HeLa cells. Taken together, these results demonstrate that the PRME extract and puerarin exert antitumor effects by inhibiting cell proliferation and inducing apoptosis of HeLa cells through both the extrinsic death receptor and the intrinsic mitochondrial pathways.

In this study, we evaluated whether the PRME extract and puerarin could improve the symptoms and inflammation associated with vaginitis. Therefore, we used a GV-infected vaginitis mouse model to determine the effects of the PRME extract and puerarin. As a result, although the PRME extract and puerarin treatment did not significantly restore the reproductive tract shortening and edema, they were visually confirmed to have been slightly restored.

MPO is an enzyme mainly expressed in neutrophils that can be used as a biochemical indicator of the degree of neutrophil infiltration and inflammation in the vaginal tissue [19]. We investigated the effects of the PRME extract and puerarin on MPO activity in the vaginal tissue of mice with GV-induced vaginitis. Our data showed that the increase in MPO activity in vaginal tissue during GV infection was reduced by the PRME extract and puerarin treatments.

GV infection significantly increased the apoptosis of vaginal epithelial cells, thereby damaging the epithelial barrier. Although exfoliation of epithelial cells is a mechanism to protect vaginal tissue by removing attached bacteria, excessive stratification can expose the underlying tissue, increasing the risk of secondary infection, including increased inflammation because of other related bacteria [20]. We analyzed the effects of PRME and puerarin on the histological changes in the vaginal tissue of GV-infected mice with vaginitis. Our results showed that the GV-infected group exhibited exfoliation of vaginal epithelial cells, an increased cornified layer, and transitional epithelium thickness, and increased infiltration of inflammatory cells compared to the normal group, whereas the PRME extract—or puerarin—treated groups showed a marked improvement in exfoliation, tissue thickness increase, and infiltration of inflammatory cells compared to the GV-infected group.

Nuclear factor-κB (NF-κB) is a key transcription factor important in regulating inflammation, and its activation promotes the production of iNOS and COX-2, which mediate inflammatory responses and modulate the levels of cytokines. COX-2 plays a role in promoting angiogenesis, cell proliferation, and inflammation, and iNOS can be used as a marker of the inflammatory process [21]. Our results showed that the PRME extract and puerarin significantly inhibited the expression of COX-2 and iNOS, which are NF-κB downstream regulators. This suggests that the PRME extract and puerarin may have an immune regulation mechanism to inhibit the activation of NF-κB, an upstream regulator of COX-2 and iNOS. These mechanisms fundamentally regulate cytokine expression in mice with GV-induced vaginitis.

Previous studies have shown that proinflammatory cytokines such as TNF-α, IL-1β, and IL-6 are increased in the vagina of women with vaginitis and are associated with the severity of vaginitis [22]. TNF-α is a crucial cytokine in the inflammatory process and plays a key role in inducing the release of other proinflammatory cytokines. IL-1β is an important component of a series of inflammatory cascades along with TNF-α in response to infection produced by various cells. IL-6 is also a cytokine that regulates inflammation. IL-10 is a potent anti-inflammatory cytokine that inhibits Th1 cytokine production, thereby suppressing inflammation. A previous study reported that intravaginal inoculation with GV induces an immune response, which increases the levels of proinflammatory cytokines in the cervicovaginal fluid, leading to an inflammatory response. Moreover, the decrease in the levels of IL-10 in a vaginitis mouse model was reversed by therapeutic treatment [23]. In this regard, we investigated the effects of PRME and puerarin on inflammatory regulation in GV-infected mice. Our results suggest that PRME and puerarin alleviate the inflammatory response in vaginitis by reducing the production of proinflammatory cytokines and elevating the levels of anti-inflammatory cytokines in GV-infected mice. Taken together, our results indicate that PRME and puerarin can alleviate vaginitis by adjusting the balance between pro- and anti-inflammatory cytokines through inflammatory mechanisms and promoting the repair of the vaginal epithelial barrier.

## 4. Materials and Methods

### 4.1. Chemical Reagents

Puerarin (purity > 98%) was obtained from Sigma–Aldrich (St. Louis, MO, USA). Cytokine enzyme-linked immunosorbent assay (ELISA) kits were purchased from R&D Systems (Minneapolis, MN, USA) and BioLegends (San Diego, CA, USA). TUNEL Apoptosis Detection Kit (DNA Fragmentation/Fluorescence Staining) and myeloperoxidase (MPO) ELISA kits were purchased from Sigma–Aldrich. Aspartate aminotransferase (AST) Assay Kit (#BM-AST-100), Alanine aminotransferase (ALT) Assay Kit (#BM-ALT-100), and Creatinine Assay Kit (#BM-CRE-100) were purchased from BIOMAX (Gurisi, Gyeonggi-do, Republic of Korea). Bicinchoninic acid (BCA) assay, Hoechst 33,342 (#H1399) kit, and Urea Nitrogen (BUN) Colorimetric Detection Kit (#EIABUN) were purchased from Thermo Scientific (Rockford, IL, USA). Specific antibodies used in western blotting analysis were purchased from Bioworld (Nanjing, China), Proteintech (Rosemont, IL, USA), or Santa Cruz Biotechnology (Dallas, TX, USA). *G. vaginalis* ATCC 14,018 (GV) was obtained from KisanBio (Seoul, Republic of Korea).

### 4.2. Preparation of Pueraria lobata (Willd.) Ohwi Root Extract

The herbal medicine used in this experiment is the roots of *P. lobata* (Willd.) Ohwi was purchased directly from the Gwangmyeongdang Oriental Medicine Pharmaceutical Company (Ulsan, Republic of Korea). The herbal medicine purchased for this research was stored in optimal environmental facilities by a pharmaceutical company specializing in herbal medicines and passed strict quality inspections by the Ministry of Food and Drug Safety. Since winter, *P. lobata* (Willd.) Ohwi stores nutrients for use during a year in its roots. The roots of *P. lobata* (Willd.) Ohwi are generally harvested in late winter to early spring. The identity of *P. lobata* (Willd.) Ohwi was established by Professor Yong-Deok Jeon at the department of oriental pharmacy, college of pharmacy, Woosuk University (Wanju, Republic of Korea). A voucher specimen (2024-WSKP07) has been deposited at the department of oriental pharmacy, Woosuk University. To prepare the methanolic extract of *P. lobata* root (PRME extract), powdered *P. lobata* root was accurately weighed (50 g), mixed with 500 mL of methanol, and refluxed for 3 h at 75 °C using Heating Mantle (Daihan Scientific, Wonju, Republic of Korea). After filtering the extract using an advantec2 paper filter (Advantec, Tokyo, Japan), the filtered PRME extract was frozen in an ultra-low temperature freezer (Thermo Scientific, Rockford, IL, USA) at −75 °C for 24 h to obtain a dry extract. Next, the frozen extract was freeze-dried in a freeze dryer (Daihan Scientific, Wonju, Republic of Korea) at −80 °C and 5 m Torr for 72 h. The methanol was completely evaporated under reduced pressure using a Digital Rotary Evaporator (LK LAB KOREA, Gyeonggi-do, Republic of Korea) and an Oilless Dry Vacuum Pump (LK LAB KOREA, Gyeonggi-do, Republic of Korea). The yield of PRME extract was 2.72%, and it was stored in a refrigerator (Thermo Scientific, Rockford, IL, USA) at 4 °C until the experiment.

### 4.3. Analytical High-Performance Liquid Chromatography (HPLC)

The HPLC conditions are listed in Table 1.

### 4.4. Cell Culture

HeLa cells (Human cervical cancer, #10002, Virus Susceptibility: poliovirus1,2,3; adenovirus 3) were purchased from the Korean Cell Line Bank (Seoul, Republic of Korea). Cells were maintained and propagated in Dulbecco’s modified Eagle’s medium (DMEM) containing 10% (*v*/*v*) heat-inactivated fetal bovine serum (FBS), 100 U/mL penicillin, and 100 μg/mL streptomycin. Cells were maintained in a humidified atmosphere of 5% CO_2_ at 37 °C.

### 4.5. Cell Viability

HeLa cells were seeded in 96-well plates at 1 × 10^4^ cells/well and cultured overnight at 37 °C for attachment. Cells were then incubated with various concentrations of the PRME extract or puerarin for 24 h. Then, 0.5 mg/mL MTT solution (20 μL) was added to each well, and the wells were incubated for an additional 4 h at 37 °C. The supernatant from each well was discarded, and the crystallized, purple-colored precipitate, formazan, was dissolved with 150 μL of dimethyl sulfoxide (DMSO). After complete dissolution, the absorbance was measured at 570 nm using a Synergy HTX Multi-Mode microplate reader (BioTek, Winooski, VT, USA). Each assay was performed in triplicate.

### 4.6. Enzyme-Linked Immunosorbent Assay (ELISA) for Cytokines Detection In Vitro and In Vivo

The concentrations of interleukin (IL)-2, IL-12, interferon (IFN)-γ, and tumor necrosis factor (TNF)-α in HeLa cells and IL-1β, IL-6, IL-10, and TNF-α levels in the mouse vaginal tissue lysates were quantified using ELISA kits according with the manufacturer’s manual. The units of measurement results for all values followed the units in the ELISA kit instructions.

### 4.7. TUNEL Assay

HeLa cells were treated with the PRME extract or puerarin at different concentrations in a 96-well plate and incubated for 48 h. Then, cells were washed with phosphate-buffered saline (PBS) and fixed in 4% paraformaldehyde solution for 30 min. To confirm apoptosis, the TUNEL Apoptosis Detection Kit (DNA Fragmentation/Fluorescence Staining) was used according to the manufacturer’s instructions. Apoptosis was visualized by confocal microscopy.

### 4.8. Hoechst 33,342 Staining

HeLa cell apoptosis was observed using a Hoechst 33,342 kit. The Hoechst staining solution was prepared by diluting the stock solution to 1:2000 in PBS. Culture medium was removed from HeLa cells, and 400 μL of the staining solution was added, which was sufficient to cover the cells. The cells were incubated for 10 min in the dark, the staining solution was removed, and the cells were washed three times in PBS. DAPI staining used 1 mg/mL stock, and the cells were washed three times in PBS. Cells with fragmented and condensed nuclei due to apoptosis were detected by fluorescence microscopy.

### 4.9. Western Blotting

Western blotting was performed as previously described [24]. Briefly, HeLa cells or vaginal tissues were lysed in cold PRO-PREP protein lysis buffer containing protease and phosphatase inhibitors for 30 min. The lysate was then centrifuged at 15,000× *g* for 25 min at 4 °C, and the supernatant was collected. After quantification of proteins using a BCA assay kit, equal amounts of protein samples (20 μg) were separated by 10% sodium dodecyl sulfate (SDS)-polyacrylamide gel electrophoresis. After transfer to polyvinylidene difluoride (PVDF) membranes, the PVDF membranes were blocked for 1 h at room temperature using 5% bovine serum albumin (BSA) in Tris-buffered saline containing 0.1% Tween 20 (TBST). Subsequently, the membranes were incubated at 4 °C overnight with primary antibodies against caspase-8, caspase-9, cyclooxygenase (Cox)-2, and inducible nitric oxide synthase (iNOS). After rinsing three times with TBST, the membranes were incubated for 2 h at room temperature with horseradish peroxidase (HRP)-conjugated secondary antibodies and washed three times with TBST and once with TBS. Bands on Western blots were detected by reacting the membranes with an ECL solution. The intensity of the protein bands was confirmed using the Davinch-Invivo imaging system (Davinch-K, Seoul, Republic of Korea) and quantified using the GelQuantNET software (V 1.7.8).

### 4.10. Animals

Specific pathogen-free female C57BL/6 mice (7 weeks, weight 20–24 g) were purchased from Samtako Bio (Osan, Republic of Korea). All mice were maintained under the controlled animal room standard conditions of temperature (21 ± 1 °C), humidity (50 ± 10%), and light (12 h light/12 h dark cycle) for 1 week before the beginning of the experiment. Mice were provided with sterilized water and food ad libitum. All procedures and management related to this animal experiment were performed in compliance with the regulations of the Animal Experiment Ethics Committee of Woosuk University (approval protocol number: WS-2024-06).

### 4.11. Preparation of G. vaginalis

*G. vaginalis* (GV), a Gram-variable staining facultative anaerobic bacterium, was cultured in a Blood Agar Base medium supplemented with 10% FBS and incubated at 37 °C in a 5% CO_2_ atmosphere for up to 48 h. For the intravaginal inoculation of mice, GV concentration was adjusted to 1 × 10^8^ CFU/mL, centrifuged at 5000 rpm for 10 min, and the pellet was suspended in sterile PBS.

### 4.12. Design of Mouse Experiments

To induce mouse vaginal infections, we referred to a previous study with some modifications [25]. We used 42 mice, which were randomly assigned to seven treatment groups (*n* = 6 per group) as follows: NOR, noninfected normal group; GV, GV-infected group; LOW, GV-infected PRME extract (80 mg/kg)/puerarin (25 mg/kg) low dose orally administered groups; HIGH, GV-infected PRME extract (160 mg/kg)/puerarin (50 mg/kg) high dose orally administered groups; and clotrimazole (CLO) (2 mg/mouse), positive control group. Before GV infection, all mice were intraperitoneally injected twice with 0.125 mg of β-estradiol-3-benzoate dissolved in sesame oil (30 µL) to induce pseudoestrus, thereby weakening the immune system. On the day of infection, 20 µL of GV (1 × 10^8^ CFU in PBS) was used to inoculate each mouse intravaginally. The control group was treated with 20 µL PBS. The treatments were resuspended in water and orally administered once daily for 14 d after infection. The normal group was administered a saline solution for the same period. On the last day, the mice were euthanized by inhaling 60 to 70% CO2 for approximately 5 min, and vaginal tissues were collected for ELISA, MPO activity analysis, histopathological analysis, and western blotting. The collected vaginal tissues were stored in a −80 °C degree freezer until subsequent experiments. The experimental schedule is shown in Figure 5A.

### 4.13. MPO Activity in Vaginal Tissue Lysates

MPO activity was measured using vaginal tissue lysates. Vaginal tissues were lysed using RIPA buffer and centrifuged at 4 °C at 12,000 rpm for 20 min. Protein was quantified using a BCA assay kit, and MPO activity was analyzed using an ELISA kit, according to the manufacturer’s instructions. MPO activity was measured using a Synergy HTX Multi-Mode microplate reader (BioTek, Winooski, VT, USA). The unit of MPO activity measurement results followed the units in the ELISA kit instructions.

### 4.14. Serum Biochemical Parameters Analysis

Blood samples were obtained from mice after euthanasia. Blood was immediately centrifuged at 3000 rpm for 10 min, and serum samples were collected. Using AST, ALT, BUN, and creatinine ELISA kits, serum biochemical parameters for liver integrity (AST and ALT) and renal function (BUN and creatinine) levels were confirmed, according to the manufacturer’s instructions. The units of measurement results for all values followed the units in the ELISA kit instructions.

### 4.15. Histological Analysis

To confirm the effects of PRME and puerarin in GV-infected mice, histopathological changes in the vaginal tissues were analyzed. The vaginal tissues of mice were excised, and the isolated vaginal tissues were fixed with 4% paraformaldehyde for 24 h and then cut into 4-μm-thick sections. Tissue sections were stained with hematoxylin for 1 min and eosin for 3 min (H&E) and visualized using a digital scanner for histological analysis. The vaginal epithelial thickness was measured randomly at five sites per tissue under a ×200 magnification light microscope equipped with a camera (Olympus CX21). The severity of vaginal epithelial lesion exfoliation and inflammation was quantified by scoring from 0 to 3 according to previous criteria (0 none, 1; minimal, 2; moderate, 3; very robust) [26].

### 4.16. Reverse Transcription Quantitative Polymerase Chain Reaction (RT-qPCR)

Total RNA was isolated from vaginal tissues using 1 mL of TRIzol solution and reverse transcribed using the Prime Super Script™ II cDNA Synthesis Kit (Takara Bio, Inc., Mountain View, CA, USA) in accordance with the manufacturer’s protocol. RT-qPCR reactions were performed for specific genes using the SYBR Green PCR Master Mix and the Applied Biosystems StepOne system (Applied Biosystems, Waltham, CA, USA) under the following thermal cycling conditions: 42 °C for 5 min, 95 °C for 15 s, and 40 cycles of 95 °C for 20 s and 60 °C for 30 s). Primers used are listed in Table 2. Glyceraldehyde 3-phosphate dehydrogenase (GAPDH) was used as the internal reference gene to quantify the relative mRNA expression.

### 4.17. Statistical Analysis

Results are presented as the mean ± SEM (standard error of the mean) of at least three independent experiments and analyzed using the Graph Pad Prism software 5.0 (San Diego, CA, USA). To compare independent variances among groups, statistical significance was determined using one-way analysis of variance (ANOVA) followed by Bonferroni’s *post hoc* analysis. The thresholds for statistical significance were set at * *p* < 0.05, ** *p* < 0.01, and *** *p* < 0.001.

## 5. Conclusions

When the immune response in our body becomes excessive, inflammation increases, leading to tissue damage or various inflammatory and chronic diseases. Therefore, in such cases, it is necessary to suppress inflammation and maintain a balanced immune response through the action of the anti-inflammatory pathway. In cervical cancer and bacterial vaginosis, inflammatory cytokines are excessively produced, stimulating the immune response. It was confirmed that treatment with PRME extract and puerarin regulates the expression of various cytokines in these conditions, thereby maintaining the balance of the immune system and preventing excessive inflammation. In addition, we demonstrated the antitumor efficacy of the PRME extract and puerarin against cervical cancer by showing an anti-proliferative effect that induces apoptosis in HeLa cells and established the anti-inflammatory efficacy of the PRME extract and puerarin by substantially improving vaginitis pathologically and biochemically in GV-infected mice. Therefore, PRME and puerarin can be used as potential adjuvants or therapeutic agents to inhibit and manage the progression of cervical cancer and alleviate vaginitis. In addition, this study is significant in suggesting the effective concentration range of the PRME extract and puerarin for these diseases.

## Figures and Tables

**Table 1:** Operating conditions of high-performance liquid chromatography (HPLC).

Parameter	Conditions	
Instrument	SCL-40	
Detector	SPD 40 at 254 nm	
Column	YMC-Pack Pro C18 (YMC, Kyoto, Japan)	
Column temperature	30 °C	
Injection volume	10 μL	
Flow rate	1 mL/min	
Mobile phase	(A) = Water	(B) = Acetonitrile
Time (min)		
0	95	5
30	83	17
40	70	30
45	75	25
55	95	5
60	100	0

**Table 2:** Primer sequences for RT-qPCR.

Gene	Forward	Reverse
mCOX-2	GAAGTCTTTGGTCTGGTGCCTG	GTCTGCTGGTTTGGAATAGTTGC
miNOS	CAGCTGGGCTGTACAAAC	CATTGGAAGTGAAGCGATTCG
GAPDH	CATGGCCTTCCGTGTTC	CCTGGTCCTCAGTGTAGC

## Data Availability

The data are contained within the article and Supplementary Materials.

## Supplementary Materials

The following supporting information can be downloaded at https://www.mdpi.com/article/10.3390/ijms26031342/s1.

## Abbreviations

BCA, Bicinchoninic acid; BV, Bacterial vaginosis; COX-2, cyclooxygenase-2; CLO, clotrimazole; Doxo, doxorubicin; DMEM, Dulbecco’s modified Eagle’s medium; DMSO, dimethyl sulfoxide; ELISA, Enzyme-linked immunosorbent assay; FBS, fetal bovine serum; GV, *Gardnerella vaginalis*; HPLC, High-Performance Liquid Chromatography; IFN-γ, interferon-γ; IL, interleukin; iNOS, inducible nitric oxide synthase; MPO, Myeloperoxidase; NF-κB, nuclear factor-κB; PBS, phosphate-buffered saline; TNF-α, tumor necrosis factor-α.
