# Supplementary material for: Methanol Extract of Pueraria lobata (Willd.) Root and Its Active Ingredient, Puerarin, Induce Apoptosis in HeLa Cells and Attenuates Bacterial Vaginosis in Gardnerella vaginalis-Infected Mice
Source: Int J Mol Sci. 2025 Feb 5;26(3):1342. doi: 10.3390/ijms26031342 (PMC11818357; doi:10.3390/ijms26031342)
Supplement: Supplementary file 1 [file ijms-26-01342-s001.zip › ijms-3397216-supplementary.pdf]

Highlights

- Puerarin, major bioactive in *Pueraria lobata* root methanolic extract (PRME extract).
- PRME extract and puerarin attenuate inflammatory cytokine levels in HeLa cells.
- PRME extract and puerarin inhibit HeLa cell proliferation by inducing apoptosis.
- PRME extract and puerarin alleviate symptoms in mice with vaginitis.
- PRME extract and puerarin suppress inflammatory markers, altering cytokine levels in mice with vaginitis.
